# A Description of Some English Appliances

**Published:** 1897-08

**Authors:** George F. Grant

**Affiliations:** Boston, Mass.


### A DESCRIPTION OF SOME ENGLISH APPLIANCES.[1]

BY GEORGE F. GRANT, D.M.D., BOSTON, MASS.

Mr. President and Gentlemen,—In October, 1895, my friend Dr. W. Booth Pearsall, of Dublin, paid me a friendly visit. Our talk drifted to a comparison of methods in use by men of our respective countries for facilitating operations of different kinds.

As an outcome of this conversation, Dr. Pearsall kindly sent

---

[1] Read before the Harvard Odontological Society, December 3, 1896.

some appliances to me which he had found useful in his practice, with a request that I give them a trial and report to him my impressions.

After a year of careful trial and experience with some of the articles, I find much pleasure in introducing them to the notice of this Society, believing that their merit fully vindicates Dr. Pearsall's claim that the American dentist has not the entire monopoly of inventive genius.

The first of these helps is a moulding-flask, an invention of Dr. Pearsall. You will readily see that, while it is simplicity itself as to construction and manipulation, its use insures the greatest accuracy in the swaging of plates upon dies cast in it. You will also observe that the whole force of the blow in swaging falls upon the apex of a cone, and at the same time less metal is required than is used in any other moulding-flask now before the profession.

The second on the list is known as the Lennox matrix. This little instrument has been especially useful to me, and I regard it a very ingenious and valuable matrix; it is the only matrix I have seen in which the band, owing to its form (a segment of a circle), must readily adapt itself to the contour idea in fillings and secure adaptation to the neck of the tooth upon which it is placed. It is also very firmly retained, and can be easily attached to all bicuspid or molar teeth if the directions as to accurate adjustment of the length of the band are faithfully observed. I have attached the matrix to a porcelain bicuspid, which, as you will perceive, presents about as difficult surfaces as are often found in the mouth; still it is firm.

Two other most ingenious and valuable devices are presented in the *collar-crown outfit* and the fusible metal base-plate. While my method of procedure in the making of collar-crowns does not permit of my employment of the whole of Mr. Lennox's device for that purpose, I am of the opinion that it can be used with the greatest satisfaction as a whole in securing perfect collars for crowns. I can, however, speak in the highest terms of the instrument designed for measurement of roots, which is a part of the outfit. I consider it invaluable for the purpose for which it is designed, and have never made a measurement by any other means since it came into my hands.

The fusible metal base-plate is an idea most skilfully developed. It can be produced in a short time,—within an hour from the taking of the impression,—and is accurate as to fit, reliable as to furnishing a sure, solid base upon which to obtain the occlusion of the teeth, under the same conditions as practically prevail in the finished piece.

The directions which accompany the system are so simple that I will not take up your time in any description or elaboration of them, simply presenting this cast with wax base-plate duplicated in fusible metal, and the mould of King's modelling composition in which it was cast. You will see that every line and feature of the cast is perfectly fitted, and, so far as I am aware, there is no more perfect, practical method of obtaining such a result in use in our profession to-day. I will add that the fusible metal lends itself to many little uses, such as an aid in the hasty adjustment of retaining appliances in orthodontia, repair of plates, attaching teeth in rubber cases, etc. The fact that the crucible can be sufficiently heated in a vessel containing boiling water, enabling me to make a die directly from the impression, makes this a very valuable addition to both operating-room and laboratory.

I had omitted the statement that all these articles were kindly forwarded by C. Ash & Son, of London, through their New York house. I presume they would be glad to forward them to any member of the profession needing them on application.

I thank you for so kindly a hearing, and will be glad to answer any question or explain any point upon request of gentlemen present.

